# A decade of hair-loss clinical trials: a registry-based analysis of studies registered on ClinicalTrials.gov

**DOI:** 10.3389/fmed.2026.1821858

**Published:** 2026-06-22

**Authors:** Alexa D. Parra-Guerra, Luis E. Sánchez-Dueñas, Mariana León Quintero-Loreto, Deyanira G. Quinoñes-Hernández, Jessica A. Moreno-Alanis, Farah K. Sevilla González, Cesar J. Ramos Cavazos, Daniela Gutiérrez Mendoza, César D. Villarreal-Villarreal, Jonathan M. Chejfec-Ciociano

**Affiliations:** 1 Research Unit, Renati Innovation, Onkimia, Guadalajara, Mexico; 2 Dermatologic Institute of Jalisco “Dr. José Barba Rubio”, University of Guadalajara, Guadalajara, Mexico; 3 Hospital San Javier, Guadalajara, Mexico; 4 Trico and Derm Clinc, San Pedro Garza García, Mexico; 5 Hospital Universitario “Dr. José Eleuterio González” de la UANL, Monterrey, Mexico; 6 AROAH Denter Dermatología, Tijuana, Mexico; 7 ITESM Monterrey, Nuevo León, México

**Keywords:** alopecia, bibliometric, meta-research, outcome measures, trial landscape

## Abstract

**Background:**

ClinicalTrials.gov captures registered hair-loss trials across the full research lifecycle, including ongoing, discontinued, and unpublished studies. We used this registry to describe how the hair-loss clinical trial landscape has evolved from 2015 to 2025, focusing on trial volume, geography, indications, intervention types, outcome assessment, and sponsorship.

**Methods:**

We conducted a descriptive cross-sectional meta-research analysis of hair-loss–related interventional studies registered on ClinicalTrials.gov (2015–2025). Trials were categorized by recruitment status, country/region, sex, and age eligibility, primary indication, intervention modality, primary outcome assessment approach, and sponsor type. We summarized distributions and temporal trends using descriptive statistics.

**Results:**

We identified 514 hair-loss trials, with registrations increasing over time, particularly after 2020. Activity was global but concentrated in select regions, especially North America, and most trials were completed or recruiting. Androgenetic alopecia and alopecia areata predominated; pharmacologic therapies were most common overall, with greater therapeutic diversity in androgenetic alopecia. Outcome reporting was heterogeneous, objective imaging was infrequent, and sponsorship was divided between industry and academic/non-profit institutions.

**Discussion:**

This registry-based analysis nevertheless delineates a rapidly expanding yet structurally uneven landscape of hair-loss clinical research, marked by geographic concentration, therapeutic clustering, and variability in outcome specification. By systematically mapping trial activity, indications, modalities, sponsorship, and completion patterns, the study reframes attention from individual therapies to the broader architecture of evidence production. These findings underscore the need for standardized outcomes, broader population representation, and closer alignment between research investment and clinical need in hair-loss disorders.

## 1 Introduction

Hair-loss disorders comprise a heterogeneous group of conditions with distinct pathophysiology and clinicopathological characteristics, including non-scarring alopecia's such as alopecia areata (AA) and androgenetic alopecia (AGA), as well as cicatricial alopecias (1). Advances in hair follicle biology, immunology, and genetics have expanded understanding of disease susceptibility and mechanisms, particularly in AA, AGA, and female pattern hair loss, contributing to increasing public and scientific interest in these conditions (1). Hair loss is a common clinical complaint associated with substantial psychological distress and reduced quality of life across diverse patient populations (2–5).

Substantial evidence indicates that hair-loss conditions are associated with impaired health-related quality of life (HRQOL). In AA, patients experience significant HRQOL impairment, particularly in mental health domains, with meta-analytic evidence demonstrating reduced scores in emotional well being, vitality, and role-emotional functioning compared with matched controls (4). Multiple generic, dermatology-specific, and AA-specific HRQOL instruments have been used, although validation studies confirming their applicability in AA remain limited (4). Similarly, AGA has been associated with trichodynia, anxiety, low self-esteem, and emotional distress, with pooled analyses showing moderate impairment in HRQOL and emotional domains, despite the absence of a consistent association with depressive symptoms (5). Factors such as hair loss severity, treatment status, and sociodemographic characteristics have been shown to influence HRQOL outcomes in AGA populations (5).

In parallel with the growing recognition of psychosocial burden, the therapeutic landscape for hair-loss disorders has expanded rapidly. For AGA, current management includes androgen-targeting and non–androgen-targeting approaches, with topical minoxidil and oral finasteride remaining the most widely used treatments, alongside dutasteride, low-dose oral minoxidil, and emerging topical or injectable formulations (3, 6). Additional modalities such as platelet-rich plasma, low-level light therapy, and device-based interventions have been explored, although heterogeneity in procedures and limited comparative evidence complicate efficacy assessment (6). In AA, traditional management has relied on topical and intralesional corticosteroids for mild disease and systemic immunosuppressants for severe cases. Still, recent advances in understanding immune pathways have led to the development of targeted therapies, including Janus kinase inhibitors, with baricitinib (2022) and ritlecitinib (2023) recently approved by the US FDA (7). Beyond pharmacologic therapies, novel drug delivery systems, including microneedling, laser-assisted delivery, radiofrequency, sonophoresis, iontophoresis, and nanoparticle-based approaches, have been developed to enhance scalp drug penetration and optimize treatment outcomes across alopecia types (2, 8). The rapid proliferation of therapeutic strategies has contributed to an increasingly complex and evolving research landscape.

Despite this expansion, the evidence base for hair-loss therapies remains fragmented. Studies vary widely in design, endpoints, follow-up duration, and methodological rigor, and establishing long-term effectiveness and safety persists challenging for many interventions (6, 8). Moreover, selective publication and incomplete reporting can limit the visibility of discontinued, negative, or early-phase studies, raising concerns about the representativeness of the published literature (1). Clinical trial registries, particularly ClinicalTrials.gov, provide a comprehensive system-level view of research activity and have transformed access to information on trial design, interventions, populations, and sponsorship over time (9). Registry-based analyses have demonstrated that many completed studies across biomedical fields fail to disclose results within several years, highlighting persistent gaps between conducted research and publicly available evidence (1). As the number and diversity of hair-loss trials continue to grow, systematic evaluation of registered clinical trials is needed to characterize trends, identify imbalances, and contextualize emerging therapies within the broader clinical research ecosystem (8, 9). Meta-research, or “research on research,” applies scientific methods to evaluate and improve how research is designed, reported, verified, evaluated, incentivized, and organized (10).

Despite the expanding volume of research in hair-loss disorders, the field lacks a comprehensive, registry-wide longitudinal analysis examining how clinical trial activity has evolved over time. To date, no study has systematically mapped the structural composition and temporal dynamics of hair-loss–related trials at a global level. The present study addresses this gap by conducting a decade-long registry-based analysis of clinical trials registered on ClinicalTrials.gov. By characterizing patterns of research activity and their evolution over time, this work provides a systems-level perspective on the contemporary hair-loss clinical research landscape and identifies areas of concentration and underrepresentation.

## 2 Methods

### 2.1 Study design and data source

We conducted a retrospective, descriptive meta-research analysis of hair-loss clinical trials registered on ClinicalTrials.gov. ClinicalTrials.gov is a publicly accessible registry maintained by the U.S. National Library of Medicine that provides protocol-level information on interventional and observational human studies conducted worldwide. The registry was used to characterize contemporary trends in hair-loss research independent of publication status or study outcomes. Analyses focused on trials registered during approximately the last decade, defined by the year of initial registration (“First Posted”).

### 2.2 Eligibility and study selection

Eligible records included interventional human clinical studies with a valid NCT identifier and at least one hair-loss–related term listed in the ClinicalTrials.gov “Conditions” field. Hair-loss relevance was defined broadly in accordance with registry-recognized terminology and included the indexed condition term alopecia and its associated synonyms (e.g., hair loss, alopecia areata, baldness, hair thinning, balding, thinning hair, scalp hair loss, scalp alopecia, hypotrichosis, effluvium, loss of hair, and related variants). This strategy was intended to capture the full spectrum of hair-loss disorders represented in the registry. No restrictions were applied by study phase, recruitment status, sponsor type, or geographic location. Records without a valid registration date were excluded from temporal analyses.

### 2.3 Registry data extraction and curation

For each eligible trial, we extracted study status, funder type, sex eligibility, age eligibility, intervention description, sponsor information, and geographic location. Sex eligibility was harmonized across trials as follows: all sexes, female-only, male-only, or not reported. Age eligibility was grouped into mutually exclusive patterns reflecting enrollment of children, adults, older adults, or combinations thereof. These variables were used to describe the demographic scope of hair-loss research.

Geographic location was derived from trial site information, standardized to the country level, and grouped into world regions. Trials conducted in more than one country were classified as multi-country studies. Temporal trends were evaluated using the year of initial registration. Annual trial counts were summarized overall and stratified by primary indication, intervention modality, funding source, and geographic region. Changes in the composition of the trial portfolio over time were assessed using proportions within each registration year.

The “Conditions” registry field was reviewed and classified according to a predefined hierarchical framework. Trials were first screened for a hair-loss signal and then assigned to one primary indication category: alopecia areata, androgenetic alopecia, telogen effluvium, scarring or cicatricial alopecias, chemotherapy-induced alopecia, or other or non-specific hair-loss disorders. When multiple condition terms were present, a single primary indication was assigned using a prespecified hierarchy prioritizing alopecia areata, followed by androgenetic alopecia, telogen effluvium, scarring alopecias, and chemotherapy-induced alopecia. Trials that could not be confidently classified were labeled unclassifiable and excluded from indication-specific analyses.

Primary outcome measures were extracted for all eligible alopecia-related interventional studies. To characterize methodological trends, we performed a structured content analysis of the primary outcome field at the trial level. Outcome descriptions were first normalized (lowercased, punctuation removed, encoding artifacts corrected) to allow systematic keyword-based classification. Each trial was then categorized according to two independent dimensions: (1) outcome construct (what was measured, for example, disease severity, hair density, hair thickness, global assessment, patient-reported outcomes, safety, or biomarker-based endpoints) and (2) measurement modality (how the outcome was assessed, for example, clinical severity scoring systems such as the Severity of Alopecia Tool, digital trichoscopy or phototrichogram-based imaging, dermoscopy, manual hair counts, standardized photography, patient-reported surveys or quality-of-life instruments, histologic or molecular assessment, or safety reporting). Trials were classified as using a given modality if any primary outcome measure contained predefined modality-specific terms. Classification was performed programmatically using an iteratively refined rule-based text classification framework informed by pilot review of registry terminology and common intervention and outcome nomenclature; ambiguous records underwent manual review before final categorization. Analyses were conducted at the trial level to avoid overcounting outcome fragments within a single study.

Intervention modality was classified using a rule-based text-mining approach applied to the ClinicalTrials.gov “Interventions” field. Intervention descriptions were converted to lowercase and screened for predefined keyword patterns corresponding to major therapeutic categories. Pharmacologic therapies were identified through drug-related terms (e.g., “drug,” “oral,” “tablet,” “capsule,” and “topical”) and specific agents commonly used in hair disorders (e.g., minoxidil, finasteride, dutasteride, spironolactone, bimatoprost, corticosteroids, methotrexate, baricitinib, ritlecitinib, deuruxolitinib, and monoclonal antibodies). Procedural or biologic interventions were defined by terms indicating injectable or biologic treatments, including platelet-rich plasma, platelet-rich fibrin, stem cell–based therapies, stromal vascular fraction, or injection-based approaches. Device-based therapies were identified by keywords such as “device,” “laser,” “light therapy,” “low-level laser therapy,” “ultrasound,” “radiofrequency,” or “microneedling.” Surgical interventions were classified using terms including “hair transplantation,” “transplant,” or “surgery,” whereas cosmeceutical or nutraceutical approaches were identified through terms such as “supplement,” “nutraceutical,” “vitamin,” “mineral,” “botanical,” “shampoo,” “conditioner,” “serum,” “lotion,” “cosmetic,” or “hair care.”

Each trial was assigned a primary intervention modality using a predefined hierarchical framework prioritizing pharmacologic, procedural or biologic, device-based, surgical, and cosmeceutical or nutraceutical approaches. Trials involving heterogeneous multimodal interventions without a dominant category were classified as other or mixed. Pharmacologic trials were further grouped into broad therapeutic subclasses for exploratory analyses. Sponsor and funder information were extracted and standardized. Funder type was categorized as industry, government or public agency, academic or non-profit organization, or not reported, and further grouped as commercial vs. non-commercial for selected analyses.

### 2.4 Statistical analysis and software

All analyses were descriptive in nature and conducted at the trial level. Categorical variables were summarized using absolute frequencies and percentages. Temporal trends were evaluated by calculating annual counts and proportions of trials stratified by condition classification, intervention modality, funding source, and outcome measurement characteristics. Proportional distributions across years were visualized using stacked bar and area plots to account for variation in annual trial volume.

No formal hypothesis testing was prespecified, as the objective of this registry-wide analysis was to descriptively characterize longitudinal patterns in the hair-loss clinical trial landscape rather than evaluate causal or comparative associations. When applicable, proportions were calculated relative to the total number of eligible trials within the specified year or subgroup. All data processing, text normalization, classification procedures, and statistical analyses were performed in R (RStudio) version 3.6.0.

### 2.5 Ethical considerations

This study analyzed publicly available, de-identified data from the ClinicalTrials.gov registry and did not involve interaction with human participants or access to individual-level identifiable information. Accordingly, it did not constitute human subjects research and was exempt from institutional review board approval. All analyses were conducted in accordance with the registry's terms of use.

## 3 Results

Between 2015 and 2025, a total of 514 hair-loss–related clinical trials were registered on ClinicalTrials.gov. Annual registrations increased steadily over the study period, rising from 29 trials in 2015 to 87 trials in 2025, with a marked acceleration after 2020. Registry data were downloaded on January 22, 2026, at which time 222 trials (43.2%) were classified as completed. An additional 159 trials (30.9%) were ongoing or had not yet begun recruitment, including 85 recruiting trials (16.5%), 36 not yet recruiting (7.0%), 31 active but not recruiting (6.0%), and 6 enrolling by invitation (1.2%). Early discontinuation was relatively common: 26 trials (5.1%) were withdrawn, and 22 (4.3%) were terminated. Eighty-three trials (16.1%) had an unknown recruitment status, while miscoded or miscellaneous status entries were rare and together accounted for less than 1% of records. Overall trial characteristics, including recruitment status, eligibility features, indications, intervention modalities, and sponsorship, are summarized in Table 1.

**Table 1:** Overall characteristics of registered hair-loss clinical trials (*N* = 514).

Characteristic	Category	No. (%) of trials
Recruitment status	Completed	222 (43.2%)
	Discontinued (withdrawn/terminated)	48 (9.3%)
	Ongoing	158 (30.7%)
	Unknown/not reported	86 (16.7%)
Sponsor type	Academic/non-profit	253 (49.2%)
	Government/public	22 (4.3%)
	Industry	237 (46.1%)
	Not reported	2 (0.4%)
Geographic region	North America	224 (43.6%)
	Europe	95 (18.5%)
	East Asia and Pacific	86 (16.7%)
	Middle East and Africa	57 (11.1%)
	South and Southeast Asia	37 (7.2%)
	Latin America	15 (2.9%)
Sex eligibility	All sexes	316 (61.5%)
	Female only	99 (19.3%)
	Male only	96 (18.7%)
	Not reported	3 (0.6%)
Age eligibility	Adults and older adults	281 (54.7%)
	Adults only	145 (28.2%)
	Children, adults, and older adults	52 (10.1%)
	Children and adults	25 (4.9%)
	Children only	9 (1.8%)
	Other	1 (0.2%)
	Not reported	1 (0.2%)
Primary clinical indication	Androgenetic alopecia	174 (33.9%)
	Alopecia areata	159 (30.9%)
	Other/non-specific hair loss	101 (19.6%)
	Unclassifiable	45 (8.8%)
	Chemotherapy-induced alopecia	18 (3.5%)
	Scarring/cicatricial alopecia	13 (2.5%)
	Telogen effluvium	4 (0.8%)
Intervention modality	Pharmacologic therapy	276 (53.7%)
	Cosmeceutical/nutraceutical	85 (16.5%)
	Device-based therapy	76 (14.8%)
	Procedural/biologic intervention	23 (4.5%)
	Surgical intervention	5 (1.0%)
	Other/mixed/unclassifiable	49 (9.5%)

**Figure 1:**
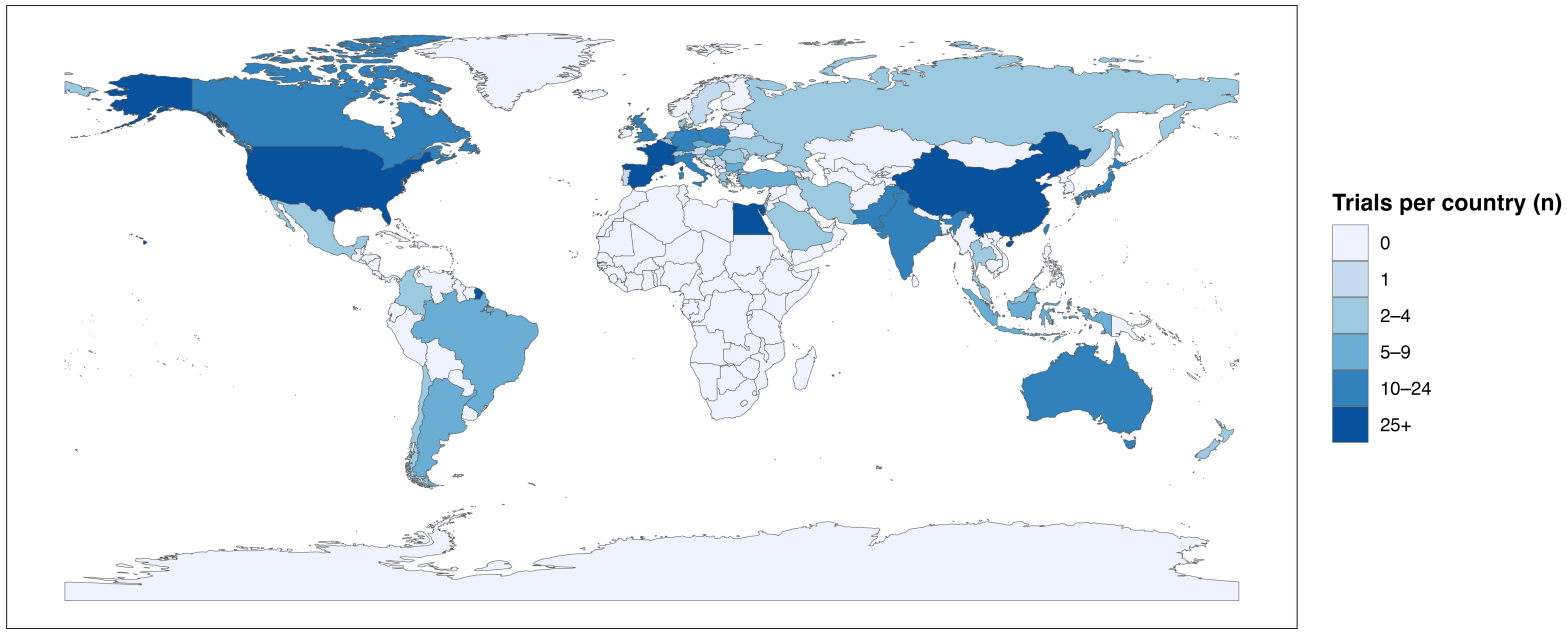
Global distribution of hair-loss clinical trials registered on ClinicalTrials.gov (2015–2025).

The 514 trials were conducted across 59 countries, demonstrating broad global participation but marked geographic concentration. The United States accounted for the largest share of trials (221; 33.0%), followed by Egypt (42; 6.3%), China (40; 6.0%), Spain (28; 4.2%), France (25; 3.7%), Canada (23; 3.4%), Germany (23; 3.4%), South Korea (22; 3.3%), and Australia (20; 3.0%). Additional countries contributing at least 10 trials included Taiwan (17), Japan (15), the United Kingdom (15), Poland (14), Pakistan (13), Italy (12), India (11), and Brazil (9). Mexico, Chile, Belgium, Hong Kong, New Zealand, and Puerto Rico each contributed four trials. The global distribution of trials is shown in Figure 1.

Most trials enrolled participants of all sexes (316; 61.5%), while 99 (19.3%) were female-only and 96 (18.7%) male-only; sex eligibility was not reported or coded as “other” in three trials (0.6%). Most studies focused on adult populations, with 281 trials (54.7%) enrolling adults and older adults and 145 (28.2%) enrolling adults only. Fifty-two trials (10.1%) included children, adults, and older adults, 25 (4.9%) included children and adults only, and nine (1.8%) were exclusively pediatric. Age eligibility was missing or uncategorizable in two trials (0.4%).

**Figure 2:**
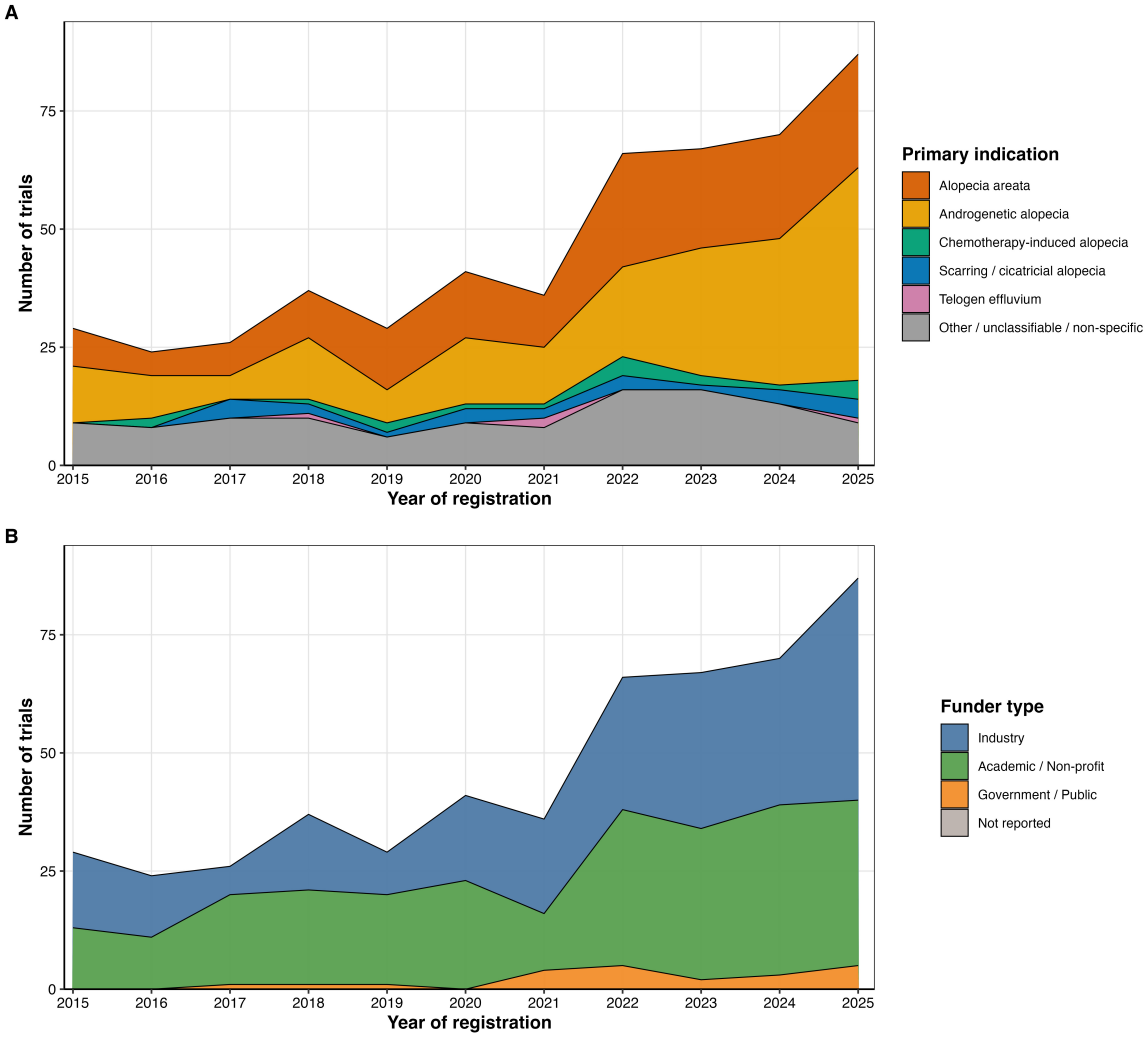
Temporal trends in hair-loss trial registrations by indication and sponsor type (2015–2025). **(A)** Stacked area plot showing the annual number of registered trials stratified by primary indication. **(B)** Stacked area plot showing the annual number of registered trials stratified by funder type (industry, academic/non-profit, government/public, and not reported). In both panels, the y-axis represents the number of trials registered per year, and the stacked areas indicate the composition of annual registrations across categories.

The most frequently studied primary indications were androgenetic alopecia and alopecia areata. Androgenetic alopecia accounted for 174 trials (33.9%), while alopecia areata accounted for 159 trials (30.9%). Other or non-specific hair-loss conditions comprised 101 trials (19.6%), and 45 trials (8.8%) were classified as unclassifiable due to insufficient or inconsistent condition descriptions. Less common indications included chemotherapy-induced alopecia (18 trials; 3.5%), scarring or cicatricial alopecias (13; 2.5%), and telogen effluvium (4; 0.8%). Only six trials (1.2%) addressed more than one specific hair-loss indication (Figure 2). Unclassifiable records largely reflected trials in which hair-related terms were absent or secondary, or where the listed conditions clearly referred to non–hair-loss diseases, such as systemic malignancies, metabolic disorders, or skin aging outcomes.

Across 514 registered alopecia interventional trials, primary outcome measurement modalities were highly heterogeneous (**Appendix Table 1**). The most frequent category was unspecified methodology, observed in 198 trials (38.5%), in which the primary outcome construct was identifiable, but the registry entry did not provide sufficient detail to confidently determine the assessment instrument or modality. Among trials with identifiable methodology, the most common modality was the use of a clinical severity score, predominantly reflecting structured clinician-administered scoring systems. Digital trichoscopy–based assessments were reported in 48 trials (9.3%), while safety reporting as a primary endpoint was observed in 44 trials (8.6%). Clinical global scales were used in 34 trials (6.6%). A subset of studies (*n* = 28, 5.5%) described hair growth or regrowth outcomes without specifying the measurement technique. Less frequently used modalities included patient-reported surveys (*n* = 25, 4.9%), manual hair counts (*n* = 24, 4.7%), dermoscopy (*n* = 9, 1.8%), and histologic or molecular endpoints (*n* = 7, 1.4%). Overall, objective imaging-based modalities remained uncommon despite increasing trial volume, and a substantial proportion of studies lacked detailed methodological specification of the primary endpoint assessment.

**Figure 3:**
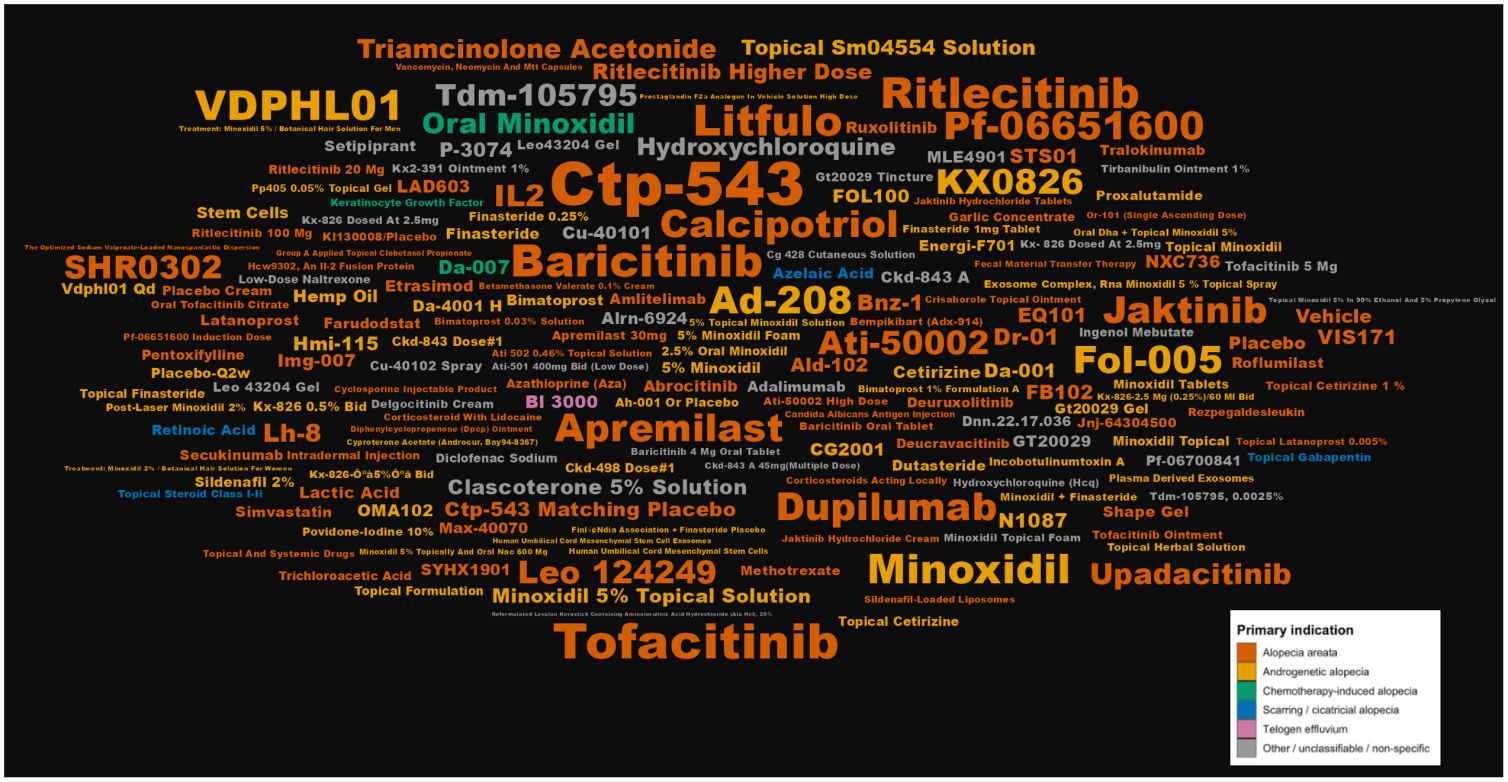
Word cloud of intervention terms across registered hair-loss trials, colored by primary indication. Word size is proportional to the frequency of each intervention term appearing in trial records. Terms are color-coded by the primary indication with which they were most commonly associated in the registry

Pharmacologic therapy was the most common primary intervention modality, used in 276 trials (53.7%). Cosmeceutical or nutraceutical approaches were employed in 85 trials (16.5%), device-based therapies in 76 trials (14.8%), and procedural or biologic interventions in 23 trials (4.5%). Surgical interventions, primarily hair transplantation, were uncommon, appearing in only 5 trials (1.0%). Forty-nine trials (9.5%) were classified as other, mixed, or unclassifiable with respect to intervention modality. To summarize the diversity of specific agents and intervention terms across indications, we visualized the most frequently represented intervention labels in a color-coded word cloud (Figure 3).

Intervention patterns varied substantially by indication. In alopecia areata, pharmacologic therapies predominated, accounting for 123 of 159 trials (77.4%), with smaller contributions from cosmeceutical or nutraceutical (5.7%), device-based (4.4%), procedural or biologic (1.3%), and surgical (0.6%) approaches; 10.7% were classified as other or mixed. In androgenetic alopecia, pharmacologic interventions accounted for exactly half of all trials (87 of 174; 50.0%), while cosmeceutical or nutraceutical interventions (20.1%), device-based therapies (14.4%), and procedural or biologic approaches (7.5%) were also prominent.

For chemotherapy-induced alopecia, device-based therapies, predominantly scalp-cooling technologies, were the leading modality (10 of 18 trials; 55.6%), followed by pharmacologic strategies (27.8%) and cosmeceutical or nutraceutical interventions (16.7%). In scarring or cicatricial alopecias, pharmacologic therapies again predominated (61.5%), with device-based therapies contributing 30.8%. More heterogeneous modality patterns were observed in the “other or non-specific hair loss” and unclassifiable categories, where pharmacologic, cosmeceutical, and device-based approaches were all represented.

When analyses were restricted to trials with definitive outcomes, completion rates differed markedly by intervention modality. Pharmacologic trials demonstrated high feasibility, with 130 of 155 trials (83.9%) completed, and cosmeceutical or nutraceutical trials showed similarly high completion rates (88.2%). In contrast, device-based therapies and procedural or biologic interventions exhibited higher discontinuation proportions, with approximately one-quarter of trials discontinued in each category. Trials classified as other or mixed had the highest discontinuation rate (35.3%).

Nearly half of all trials were sponsored by academic or non-profit institutions (253; 49.2%), with a similar proportion sponsored by industry (237; 46.1%). Government or public agencies funded 22 trials (4.3%), while the funder type was not reported in two trials (0.4%). Sponsorship was highly fragmented, with 295 distinct sponsor entities represented. Two sponsors were tied as the most active, each contributing 15 trials, but beyond these, contributions were dispersed across many organizations. Among industry-funded studies, a modest concentration was observed, with the 15 most active industry sponsors accounting for 35.5% of industry-funded trials.

## 4 Discussion

Over the past decade, the clinical trial landscape for hair-loss disorders has expanded substantially, particularly after 2020, likely reflecting increasing therapeutic innovation, industry engagement, and recognition of the clinical and psychosocial burden associated with these conditions. However, increasing trial volume does not necessarily translate into greater methodological consistency or higher-quality evidence generation. Despite this growth, several structural features persist that may affect evidence quality and clinical translation, including geographic concentration of trials, predominance of androgenetic alopecia and alopecia areata, underrepresentation of telogen effluvium and cicatricial alopecias, and heterogeneous, frequently under-specified outcome assessments that limit comparability across studies and complicate evidence synthesis. Collectively, these findings underscore the need for greater endpoint transparency, broader population representation, and more standardized approaches to outcome assessment to support clinically meaningful and generalizable evidence generation.

The predominance of trials in androgenetic alopecia and alopecia areata reflects a broader pattern in which clinical research concentrates within a subset of conditions, and research intensity may not align with population health needs or neglected disease areas (11). Meta-research has similarly shown that topic clustering and redundancy can arise when new studies are initiated without systematic consideration of existing evidence, contributing to avoidable research waste and constraining cumulative knowledge building (12, 13). In hair loss, this concentration plausibly tracks the maturity of therapeutic pipelines and the feasibility of recruiting well-defined populations, whereas more heterogeneous disorders remain relatively underrepresented. Such an imbalance risks perpetuating gaps in validated outcomes and evidence-based management for conditions that may carry substantial patient impact despite lower trial volume.

Defining outcomes is a central methodological challenge in alopecia trials, as treatment effects are often modest, measurement techniques and assessors vary, and clinical relevance depends on endpoints that are valid, reproducible, and meaningful to both patients and clinicians. Inconsistent outcome selection and incomplete reporting of measurement instruments across medicine reduce comparability between trials, complicate meta-analyses, and contribute to research waste, even when individual studies are well designed (14). Similar challenges in alopecia research may limit cumulative evidence generation and reduce the feasibility of robust cross-study synthesis. These issues have led to the development of reporting and transparency frameworks such as CONSORT and SPIRIT, which promote harmonized outcome sets to support cumulative evidence generation (15). In alopecia research, where clinician-assessed severity, objective assessment techniques, and patient-reported outcomes each capture different aspects of benefit, consensus on outcome domains and explicit reporting of measurement instruments are essential for robust inference and clinically interpretable recommendations (16, 17).

Patterns of intervention provide further insight into evidence generation. Pharmacologic therapies constitute the majority of registered trials across indications, whereas device-based, procedural, and biologic approaches account for smaller yet significant proportions. This predominance may reflect the relative regulatory maturity, scalability, and commercial feasibility of pharmacologic development pathways compared with procedural and device-based interventions. This distribution aligns with broader registry trends, where drug trials remain the predominant research modality (11). Registry-based analyses are particularly effective for capturing this diversity at the ecosystem level, including early-phase and exploratory studies that may not yet appear in the published literature (12).

The geographic concentration of hair loss trials in North America, Europe, and East Asia reflects patterns observed in dermatologic and biomedical research, where trial activity is disproportionately located in high-income regions (11, 18). This concentration may limit the generalizability of findings to populations in low- and middle-income countries and influence which patient groups and healthcare contexts are represented in the evidence base (18). This imbalance may also contribute to underrepresentation of populations with different genetic backgrounds, hair phenotypes, sociocultural perceptions of hair loss, and healthcare access patterns, potentially limiting the external validity and global applicability of emerging therapeutic evidence. Although this study did not assess disease burden or access to care, mapping the locations of trials remains essential for understanding the structure of the evidence base.

Participant eligibility patterns also warrant consideration. Most trials targeted adult populations, with relatively few studies restricted to children. Prior analyses on ClinicalTrials.gov indicate that demographic representation varies across disease categories and study designs, with persistent underrepresentation of certain populations across medical fields (19). In dermatology, underrepresentation of racial and ethnic minority groups has been documented even in recent trials, raising concerns about the applicability of trial findings to diverse patient populations (12). While our analysis characterized eligibility rather than enrollment, registry-based assessment of target populations provides a foundation for subsequent evaluations of representativeness in hair loss research.

Trial completion and discontinuation patterns are likewise informative. Meta-research across medical disciplines has shown that a substantial proportion of randomized clinical trials are discontinued prematurely, often due to recruitment limitations. These findings further reinforce that trial registration alone does not guarantee study completion or dissemination of results (20–22). Our findings of differential completion by intervention category underscore that feasibility may vary systematically by modality, which can influence which approaches ultimately contribute to the accessible evidence base independent of clinical interest or biological rationale (20–22).

Sponsorship of hair loss trials was divided almost equally between academic or non-profit institutions and industry. Meta-research indicates that sponsor type may be associated with differences in trial discontinuation and dissemination, although these effects vary by field and context (20, 22). Industry-academia collaboration remains a key mechanism for therapeutic development, leveraging infrastructure, resources, academic expertise, and access to patient populations (23). In this registry-based analysis, sponsorship patterns primarily offer descriptive insight into the organizational structure of hair loss research, rather than indicating differences in scientific quality.

Methodologically, this study highlights the value of registry-based meta-research for characterizing how evidence generation in hair-loss disorders evolves over time. Trial registries enable longitudinal assessment of trial activity, indications, interventions, sponsorship, and target populations, including studies that are discontinued or unpublished (12, 24). They also provide visibility into areas of active investigation that may remain underrepresented in the published literature. However, registry-based analyses remain dependent on the accuracy, completeness, and consistency of submitted entries (20–22, 24). Consequently, this descriptive study does not evaluate treatment efficacy, study quality, or causal relationships, but instead provides a system-level overview of evidence generation and structural gaps that may hinder synthesis and translation. Findings may additionally be affected by missing, outdated, or inconsistently coded registry fields, particularly for conditions, interventions, recruitment status, and sponsorship (11, 20–22). Some degree of misclassification remains possible for heterogeneous or insufficiently detailed entries involving non-specific conditions or mixed intervention modalities. The cross-sectional design does not capture subsequent status changes or later dissemination after data extraction, and eligibility criteria served as proxies for population inclusion rather than actual enrollment. No formal quality assessment of individual trials was performed, and reliance on ClinicalTrials.gov may underrepresent studies registered elsewhere or conducted in settings where registration in this registry is uncommon.

This registry-based analysis delineates a rapidly expanding yet structurally uneven landscape of hair-loss clinical research, characterized by geographic concentration, therapeutic clustering within a limited set of indications, heterogeneous intervention strategies, and ongoing variability in outcome specification. By systematically mapping trial activity, indications, modalities, sponsorship patterns, eligibility criteria, and completion trajectories, this study shifts attention from isolated therapeutic advances to the architecture of evidence production itself. These findings provide essential context for interpreting the current evidence base and underscore the need for more standardized outcomes, broader population representation, and improved reporting practices. From a practical perspective, these findings may help guide future trial design, encourage greater harmonization of outcome measures, and support more equitable alignment of research investment with underrepresented hair-loss conditions and populations. Collectively, these findings provide a framework for future meta-research evaluating whether therapeutic innovation, funding patterns, and evidence generation are proportionately aligned with unmet clinical needs across hair-loss disorders (12, 24).

## Data Availability

The raw data supporting the conclusions of this article will be made available by the authors, without undue reservation.
