# Supplementary material for: A decade of hair-loss clinical trials: a registry-based analysis of studies registered on ClinicalTrials.gov
Source: Front Med (Lausanne). 2026 Jun 22;13:1821858. doi: 10.3389/fmed.2026.1821858 (PMC13335679; doi:10.3389/fmed.2026.1821858)
Supplement: Supplementary file 1 [file Table_1.docx]

Supplementary Material

# Supplementary Tables

**Supplementary Table 1. Distribution of Primary Outcome Measurement Modalities in Alopecia Clinical Trials (N = 514)**

| **Measurement Modality** | **n** | **% of Total Trials** |
| --- | --- | --- |
| Clinical Severity Score | 97 | 18.9 |
| Digital Trichoscopy | 48 | 9.3 |
| Safety Reporting | 44 | 8.6 |
| Clinical Global Scale | 34 | 6.6 |
| Hair Growth (Method Unspecified) | 28 | 5.5 |
| Patient Survey | 25 | 4.9 |
| Manual Hair Count | 24 | 4.7 |
| Dermoscopy | 9 | 1.8 |
| Histology / Molecular | 7 | 1.4 |
| Unspecified | 198 | 38.5 |

**Table legend:**Primary outcome measurement modalities across 514 registered alopecia interventional trials. Percentages are calculated relative to the total number of trials. “Clinical Severity Score” includes structured clinician-administered scales (e.g., Severity of Alopecia Tool). “Digital Trichoscopy” includes phototrichogram- and image-based scalp analysis systems. “Clinical Global Scale” refers to investigator-rated global improvement or severity assessments. “Patient Survey” includes quality-of-life instruments and patient-reported outcome measures. “Manual Hair Count” includes target area or hairs-per-area counting methods. “Dermoscopy” refers to dermoscopic assessment not otherwise classified as digital trichoscopy. “Histology / Molecular” includes biopsy-based or molecular endpoints. “Hair Growth (Method Unspecified)” denotes trials describing hair growth outcomes without specifying measurement technique. “Unspecified” indicates trials in which the primary outcome construct was reported without sufficient methodological detail to determine the assessment modality.

# Supplementary Figures

**Supplementary Figure S1. Indication composition of annual registrations (within-year share, 2015–2025).**

**
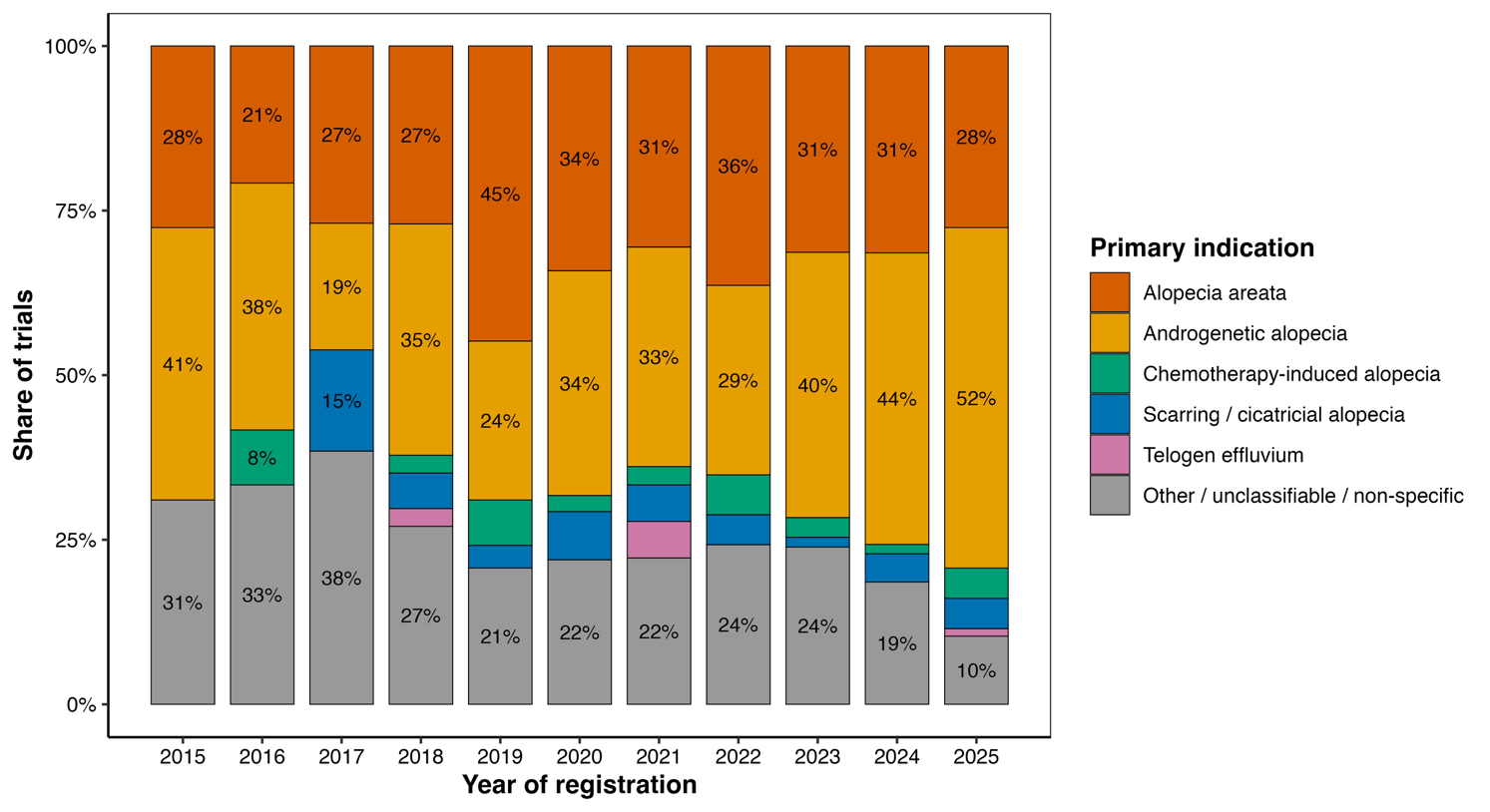
**

**Supplementary Figure S2. Annual trial registrations by sponsor type (counts, 2015–2025).**

**
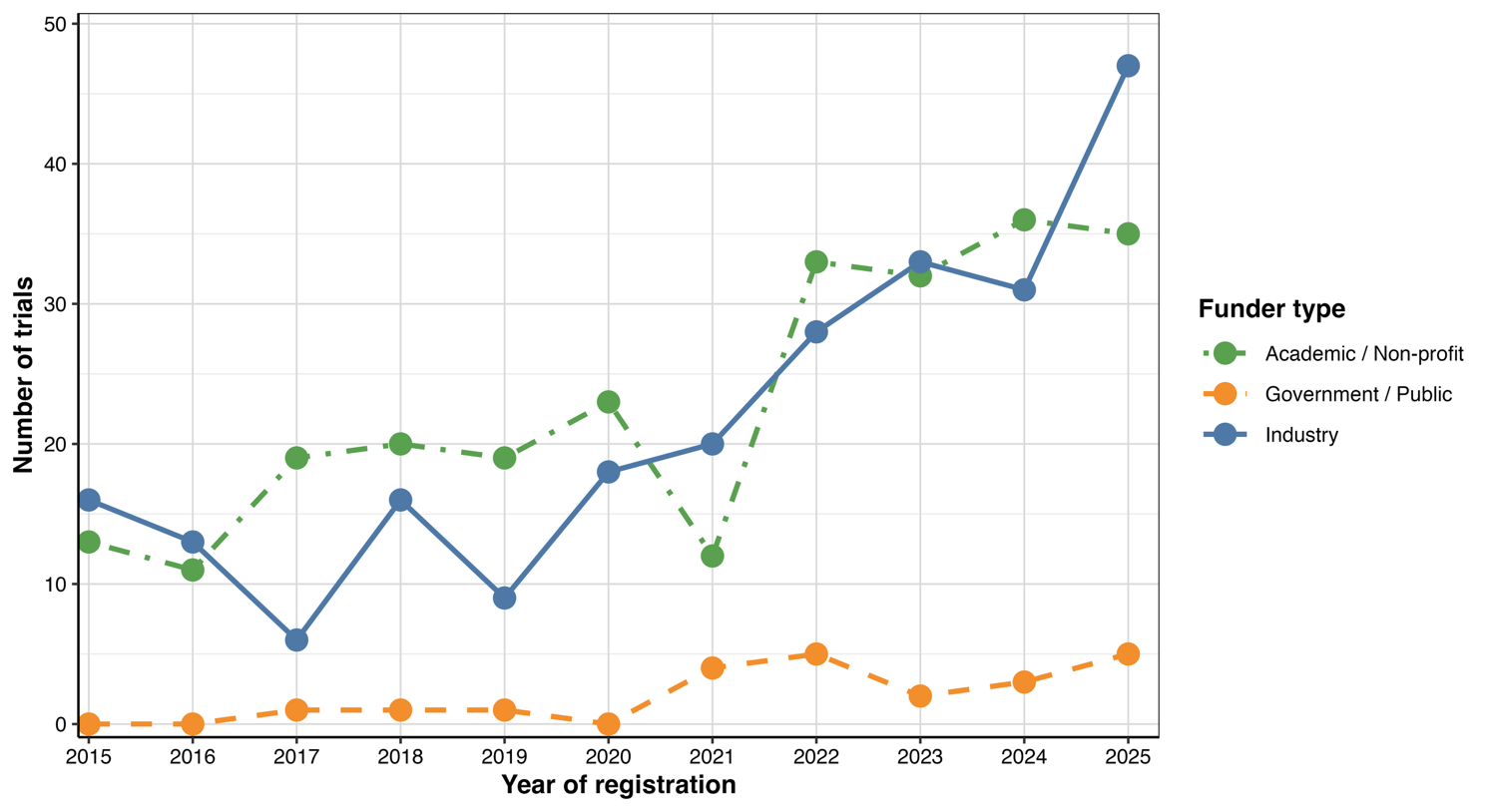
**

**Supplementary Figure S3. Regional distribution of registered trials by indication.**


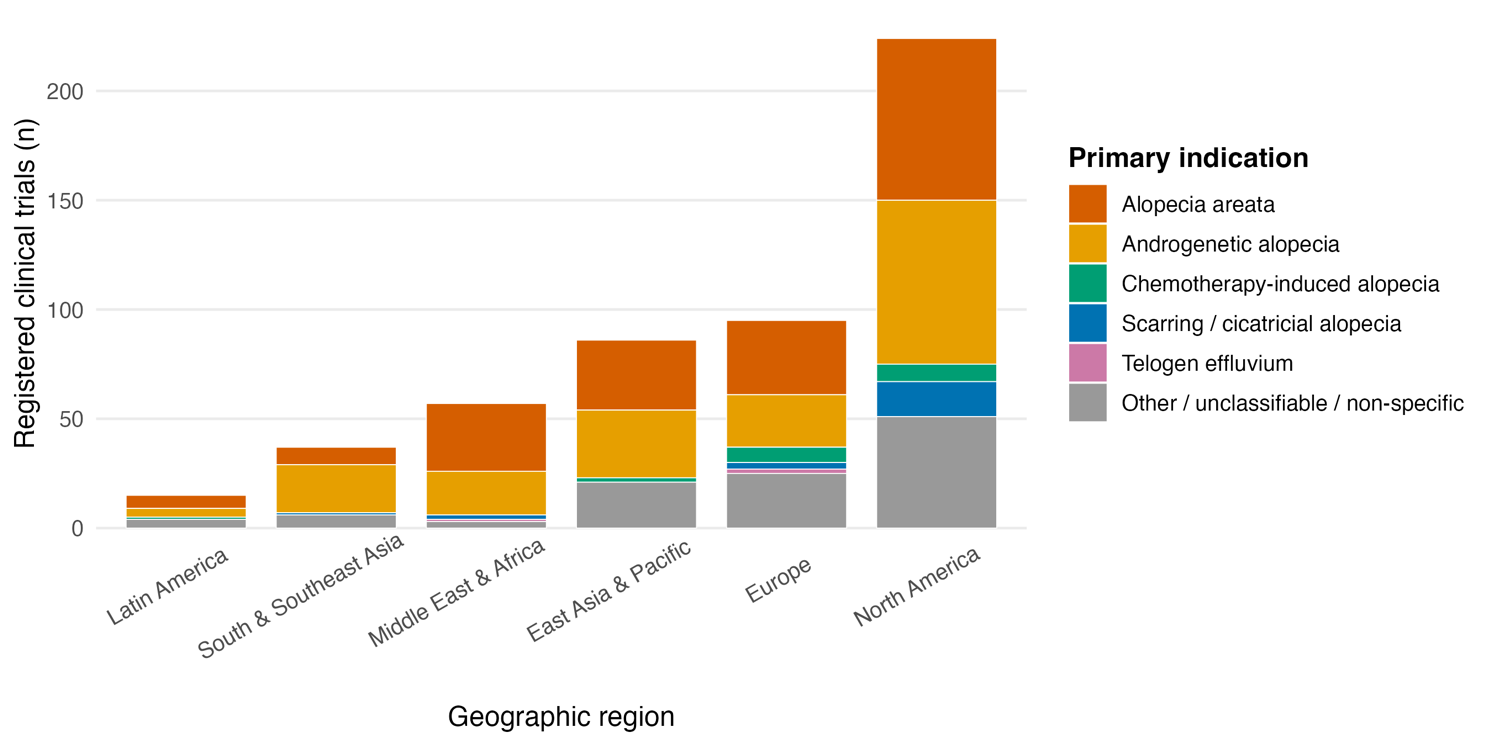


**Supplementary Figure S4. Sponsor type trends within alopecia areata and androgenetic alopecia.**

**
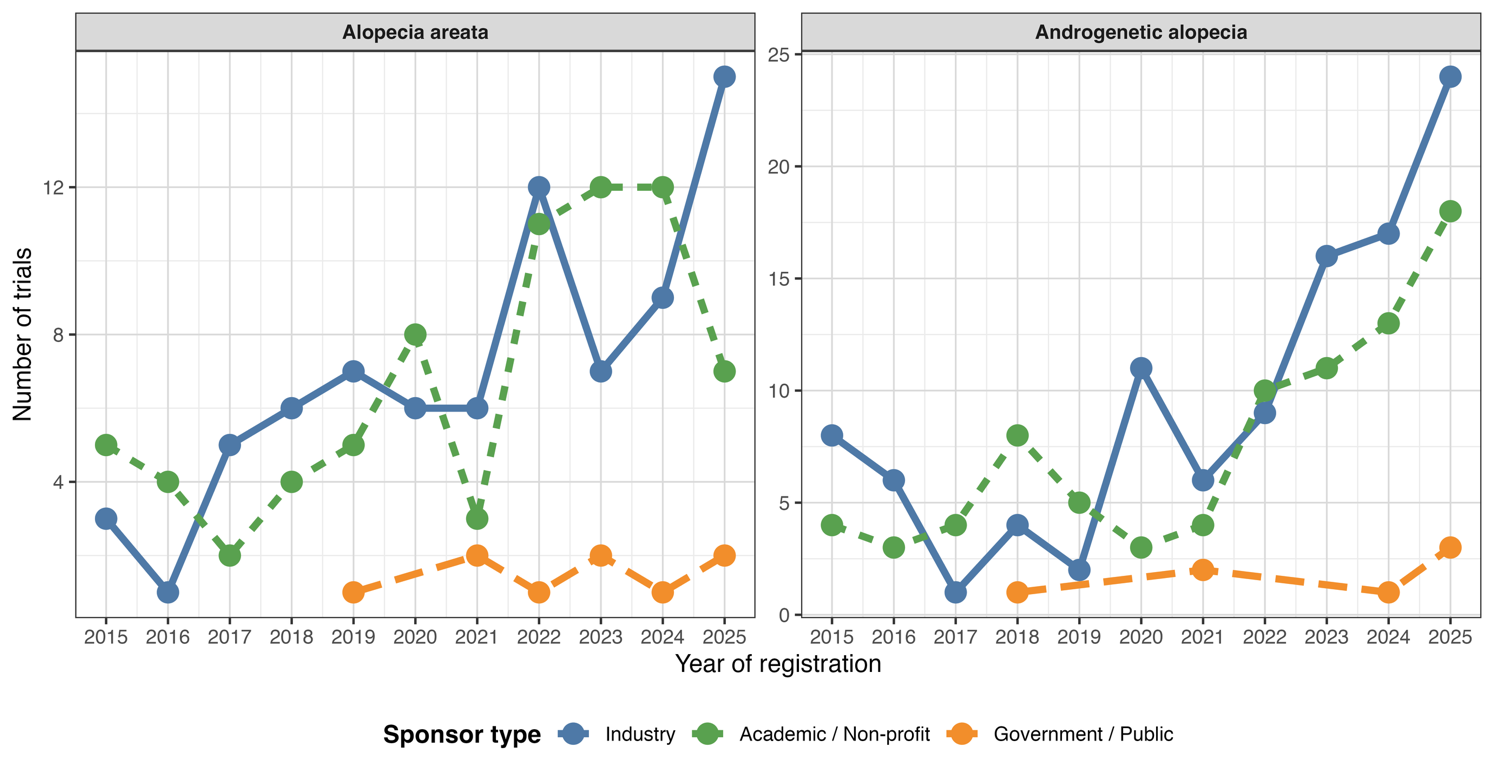
**

**Supplementary Figure S5. Indication-specific intervention modality profiles (within-indication percentages).**


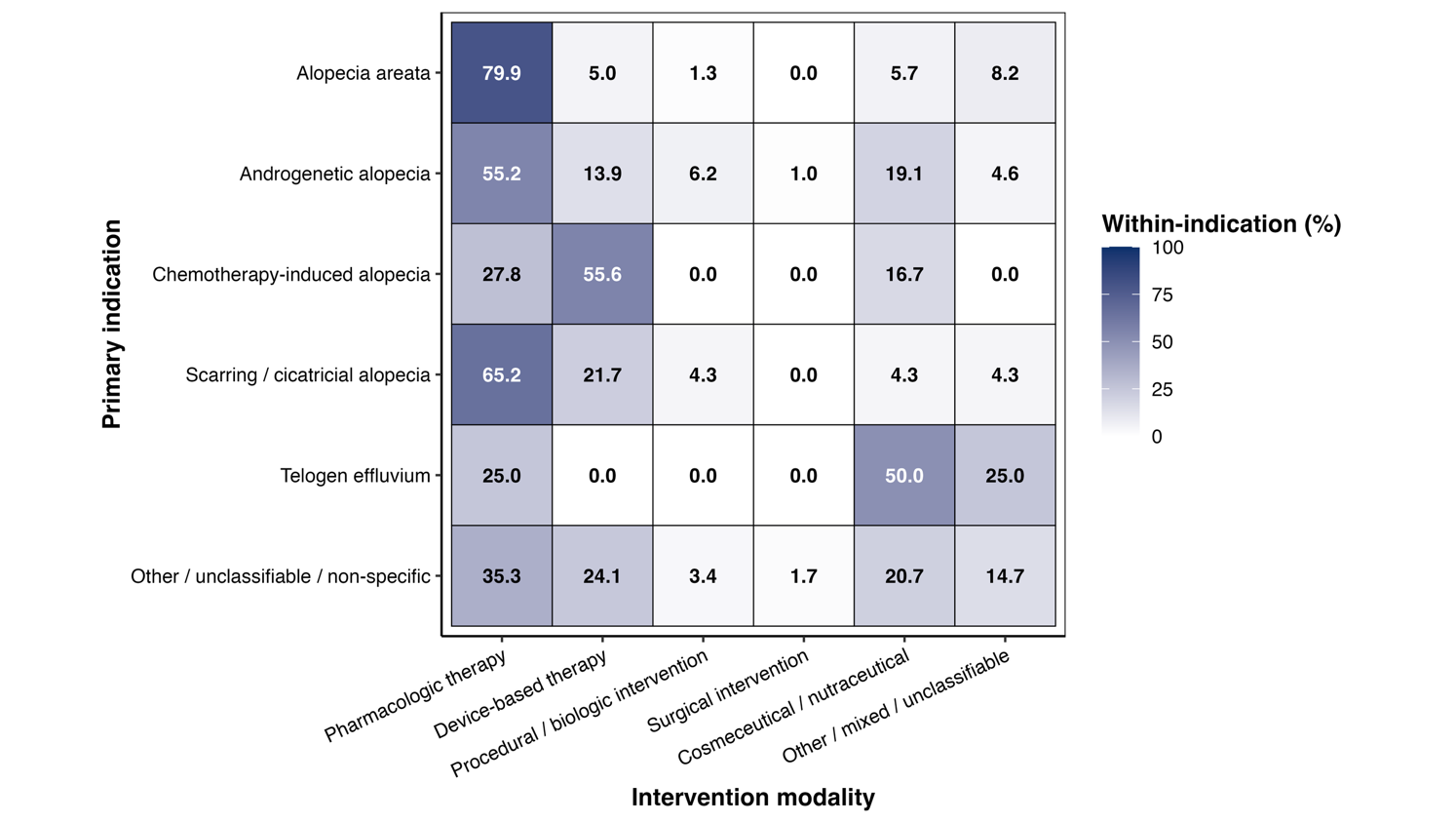


**Legend:** Heatmap showing the distribution of intervention modality within each primary indication. Each cell displays the percentage of trials for a given indication (row) assigned to a given intervention modality (column); rows sum to 100%. Darker shading indicates higher within-indication percentages.
